# An Investigation of Healthcare Professionals’ Motivation in Public and Mission Hospitals in Meru County, Kenya

**DOI:** 10.3390/healthcare8040530

**Published:** 2020-12-02

**Authors:** Rose Nabi Deborah Karimi Muthuri, Flavia Senkubuge, Charles Hongoro

**Affiliations:** 1 School of Health Systems and Public Health (SHSPH), Faculty of Health Sciences, University of Pretoria, Pretoria 0028, South Africa; flavia.senkubuge@up.ac.za (F.S.); chongoro@hsrc.ac.za (C.H.); 2 Developmental, Capable and Ethical State Division, Human Sciences Research Council (HSRC), Pretoria 0001, South Africa; 3 Faculty of Science, Tshwane University of Technology, Pretoria 0183, South Africa; 4 Faculty of Health Sciences, Fort Hare University, Alice 5700, South Africa

**Keywords:** motivation, healthcare professionals, self-determination theory, health systems strengthening, Kenya

## Abstract

Strengthening health systems in developing countries such as Kenya is required to achieve the third United Nations’ Sustainable Development Goal of health for all, at all ages. However, Kenya is experiencing a “brain drain” and a critical shortage of healthcare professionals. There is a need to identify the factors that motivate healthcare workers to work in the health sector in rural and marginalized areas. This cross-sectional study aims to investigate the factors associated with the level and types of motivation among healthcare professionals in public and mission hospitals in Meru county, Kenya. Data were collected from 24 public and mission hospitals using a self-administered structured questionnaire. A total of 553 healthcare professionals participated in this study; 78.48% from public hospitals and 21.52% from mission hospitals. Hospital ownership was statistically nonsignificant in healthcare professionals’ overall motivation (*p* > 0.05). The results showed that sociodemographic and work-environment factors explained 29.95% of the variation in overall motivation scores among participants. Findings indicate there are more similarities than disparities among healthcare professionals’ motivation factors, regardless of hospital ownership; therefore, motivation strategies should be developed and applied in both public and private not-for-profit hospitals to ensure an effective healthcare workforce and strengthen healthcare systems in Kenya.

## 1. Introduction

Strengthening healthcare systems in developing countries is required to achieve the third United Nations Sustainable Development Goal (SDG3). The SDG3 aims to “Ensure healthy lives and promote well-being for all, at all ages” [1] (p. 14). Specifically, Target 3C underscores the necessity for countries to develop, train, recruit, and retain a healthcare workforce to increase healthcare-worker density and improve their distribution to promote equitable healthcare [2]. Hence, healthcare-workforce strengthening is paramount to ensure stronger healthcare systems. To facilitate the achievement of Target 3C, the 64th World Health Assembly (WHA) passed a resolution on “Health Workforce Strengthening” (WHA 64.6) in 2011 [3]. The resolution recognizes the healthcare workforce as a significant component of healthcare systems and underscores the importance of enhancing their well-being to strengthen national healthcare systems and improve healthcare outcomes [3]. Concerning motivation, the WHA urged member states “to develop strategies and policies to increase the availability of motivated and skilled workers in remote and rural areas…” [3] (p. 3). 

Kenya is among the developing countries with suboptimally functioning healthcare systems [4]. One of the main challenges contributing to weak healthcare systems in developing countries is the critical shortage of skilled healthcare professionals, which is a significant demotivator [5]. The challenges that healthcare professionals continually face deplete their motivation to stay in the Kenyan health system, as evidenced by frequent strikes by these professionals [6,7,8]. Recommendations have been made to identify what determines healthcare professionals’ motivation to work in the healthcare sector as a way of bridging the knowledge gap related to long-term issues such as the healthcare-related costs of brain drain [9]. 

Brain drain, in this case, involves healthcare professionals emigrating from Africa to developed countries or internally from rural to urban areas [9], resulting in healthcare-related costs due to a critical shortage of healthcare professionals and their poor distribution [9]. When a medically trained doctor or nurse emigrates, Kenya loses at least USD1,854,667 and USD1,213,463, respectively, in returns from the investment made in them [9]. A study revealed that 72% of 404 healthcare workers (both technical and nontechnical) would like a job outside Kenya [10]. According to Ibrahim, cultural differences regarding modernity exist between Western societies and African nations [11]. In Western societies, modernity promotes individuality, but in African societies, it reinforces a stronger reliance on ties to family and communities of origin [11]. This insight is directly related to healthcare professionals’ motivation to emigrate from Africa to developed countries or internally from rural to urban areas. Such relocation offers a means to support their families, dependents, and communities of origin, regardless of the blurred boundaries between collectivism and individualism presented by modernity [11]. However, achieving universal health coverage (UHC) and SDG3 requires adequate, competent, and optimally-motivated healthcare professionals who are well distributed across the country [3,12]. This brain drain is a threat to the resilience of the Kenyan healthcare system and is due to push factors such as poor work conditions, work overload, delayed pay, low income, and prolonged strikes that deplete healthcare professionals’ motivation to work in Kenya [13].

Motivation in the workplace refers to the degree to which an individual willingly strives and maintains effort toward accomplishing organizational goals [14]. The more motivated healthcare professionals are, the better the quality of healthcare service delivery [12]. However, the opposite has been reported in several healthcare facilities in East African countries such as Kenya, necessitating the improvement of poor work environment/conditions, which is a demotivator among healthcare professionals [5].

The work environment entails the physical surroundings in which healthcare professionals deliver their services; in this case, hospitals. A healthy, safe, supportive, and positive-practice-enabling work environment is a major motivating factor among healthcare professionals [3,12,15]. In 2014, a Kenyan study reported that the work environment in private hospitals was rated higher than that in public hospitals [10]. Healthcare professionals are motivated to move from public hospitals to nongovernmental hospitals and other countries due to the better work environment in the latter choices [10]. The work environment in hospitals is multifaceted. In this study, our focus is on the physical work environment, regarding hygiene, water and sanitation, and occupational health and safety.

Both the public and private not-for-profit health sectors play important roles within a national healthcare system and healthcare development [16]. In Kenya, there are more skilled healthcare professionals in urban areas than rural areas [10], and more studies have been done in public hospitals than in private ones; thus, this study aims to contribute to filling the knowledge gap in both public and mission (private not-for-profit) hospitals. While the national government runs public hospitals, faith-based mission hospitals significantly contribute to healthcare service delivery in rural areas.

The primary aim of this study is to investigate the factors associated with the level and types of motivation among healthcare professionals in public and mission hospitals in Meru county, Kenya. The research questions we aim to answer are the following:

1. What are the most important and strongly supported motivational subscales among healthcare professionals?
2. What are the intrinsic and extrinsic motivators comprising healthcare professionals’ overall motivation?
3. Is there a statistically significant relationship between healthcare professionals’ overall motivation and hospital ownership?
4. What are the statistically significant differences between the mean ratings of motivational outcomes in public and mission hospitals?

According to the World Health Organization (WHO) Regional Committee for Africa resolution AFR/RC67/11, “The vision is to accelerate progress toward achieving UHC in member states by ensuring universal access to skilled and motivated health workers” [17] (p. 7). This resolution demonstrates the need for healthcare workforce strengthening to achieve UHC and SDG3. The current study will contribute to the existing literature on the motivating factors for the healthcare workforce in public and private not-for-profit healthcare sectors. This information is vital for healthcare policymakers and managers to develop and implement pertinent evidence-based policies and strategies aimed at promoting healthcare professionals’ motivation and well-being. 

### Theoretical Framework

In the mid-1980s, Deci and Ryan [18] developed an empirically-based self-determination theory (SDT). SDT specifically argues that two motivational types, intrinsic and extrinsic motivation, define the origin and strength of motivation [19,20]. These types of motivation significantly contribute to higher volition, more effective performance, and long-term persistence, especially when doing complex tasks [19,20]. Individuals are said to be intrinsically motivated when they do something based on their own interests and enjoyment [21]. Extrinsically-motivated individuals do something for reasons other than inherent satisfaction [21]. SDT argues that motivation requires a supportive environment that addresses three needs: autonomy, competence, and relatedness [22]. This stems from the idea that human beings are motivated to achieve psychological growth and integration through learning, mastery, and connection with others [21]. A complete lack of motivation is known as amotivation. Amotivation is detrimental to the workplace because it is negatively associated with performance, engagement, learning, and well-being [21]. The theory highlights the fact that the type of motivation behind actions is as important as the opposing forces that could inhibit the intended actions. SDT has been applied in multiple settings, including healthcare in Burkina Faso [23], Malawi [24], and Canada [25], among others. Thus far, there is no existing study that has applied SDT when investigating healthcare professionals’ motivation in Kenya. 

## 2. Materials and Methods 

### 2.1. Study Setting and Sample 

This study was carried out in Meru county, on the eastern slopes of Mount Kenya, which is one of 47 counties in Kenya. As of July 2020, Meru county had a total population of about 1,545,714 people [26] and a population growth rate of 2.1% per year [27]. It is mainly a rural area that relies on agriculture [27]. This study focuses on Meru county because there has been no similar research in this locality and most problems related to critical shortages of healthcare professionals are in rural areas. Most rural hospitals in Kenya are public or private not-for-profit (mission) hospitals, as opposed to private for-profit hospitals; thus, we focused on public and mission hospitals.

The county healthcare system consists of 183 health facilities, of which 63.4% are public, 24.0% are faith-based (mission), 1.6% are nongovernmental organizations, and 11.0% are private for-profit [28]. The healthcare referral system in the county is organized into five levels. These are community health workers who offer primary healthcare at Level 1, dispensaries at Level 2, healthcare centers at Level 3, subcounty (previously district) hospitals at Level 4, and the county teaching and referral hospital at Level 5 [27,29]. In 2018, the human resources for healthcare (HRH) across all cadres in Meru county amounted to 1872 healthcare professionals [27]. This study focuses on Levels 4 and 5 health facilities because they are referral hospitals. Being at the top of primary healthcare, the professionals who work there supervise and provide guidelines to all lower levels in the healthcare system [30]. In Meru county, there are 23 Level 4 hospitals and one Level 5 hospital [27]. 

This research was conducted in the 24 public and mission (faith-based) hospitals in Meru county. Simple random sampling was done among healthcare professionals in the hospitals, excluding support staff. At the time of the study, the total population of public and mission healthcare professionals eligible to participate as per our criteria was *N* = 954. 

A structured self-administered questionnaire was randomly distributed to healthcare professionals of different cadres at the hospitals. The professionals included physician and specialist doctors, clinical officers, nursing personnel, dentistry personnel, pharmaceutical personnel, medical laboratory scientists, nutritionists, public health specialists, mental health specialists, physiotherapists, radiologists, and health records officers. Participants were presented with an informed consent document, which all participants voluntarily signed before completing the questionnaire. In total, the overall response rate was 97.7%, with a total of 566 questionnaires returned. Thirteen of the returned questionnaires were excluded because 50% or more of the answers were not completed. Thus, our final sample size was 553 healthcare professionals. 

### 2.2. Data Collection

The data were collected in June and July 2020, during the COVID-19 global pandemic. However, during that time, Meru county only had 16 reported COVID-19 cases as of 30 June and only 32 reported cases at the end of July in a population of 1,545,714 individuals [23,28]. Therefore, the data were collected when the number of COVID-19 cases was low, and the pandemic did not adversely influence the data collection period of this study.

Mbindyo et al. developed a 23-item self-administered questionnaire to measure motivation based on seven work-motivation constructs: general motivation, burnout, job satisfaction, intrinsic job satisfaction, organization commitment, conscientiousness, and timeliness and attendance [31]. The items are scored on a Likert scale with a range from 1 to 5, where 1 signifies “strongly disagree” and 5 denotes “strongly agree” for positively worded items. About 40% of the items are negatively worded to avoid response-set bias. For negatively-worded questions, the coding is reversed, with 1 signifying “strongly agree” and 5 signifying “strongly disagree” [31]. Collectively, the scores from the seven underlying constructs reveal the overall motivation among the participants [31].

The data collection instrument has been validated and found useful in Kenya [31,32], Ethiopia, [33] and Zambia [34]. According to Hair et al. [35], if the Cronbach’s alpha is higher than 0.70 on a scale of 0 to 1, then the tool is reliable. Previously, the internal consistency of the scale had been assured with a Cronbach’s alpha of 0.75 and 0.70 [31,33]. Internal consistency in this study was assured with a Cronbach’s alpha of 0.85, showing that the scale was reliable in measuring motivation among the participants (see Table A1 for details of the internal consistency of the motivational subscales). 

Sociodemographic and work environment sections were also included in the questionnaire, which was pretested among healthcare professionals. Pretesting focused on assessing the relevance and comprehensibility of the items. Contextualized application of the sociodemographic and work-environment aspects was applied as per the Kenyan setting. 

### 2.3. Data Analysis 

Data entry, transformation, and analysis were performed using Excel (Microsoft, Redmond, WA, USA) and STATA 15.1^®^ (StataCorp., College Station, TX, USA). This research used descriptive statistics (frequencies, means, medians, and standard deviations), the relative importance index (RII), mean absolute deviation from the median (MADM), and multivariate regression analysis. The descriptive statistics describe the data precisely using percentages, frequency distributions, and measures of central tendency. 

The relative importance of motivational outcomes was measured using RII. Subsequently, a ranking using RIIs of the motivational outcomes was performed. The RII formula used to analyze the relative index is as follows:

$$RII=\sum \frac{W}{A\times N}$$

where *W* is the weighting given by each participant on a 5-point Likert scale, in which 1 indicates low motivation and 5 indicates higher motivation; *A* is the highest weight, which was 5; *N* is the total number of participants. RII ranges between 0 and 1, where values between (0.8 ≤ *RII* ≤ 1) are high, (0.6 ≤ *RII* ≤ 0.8) are high–medium, (0.4 ≤ *RII* ≤ 0.6) are medium, (0.2 ≤ *RII* ≤ 0.4) are medium–low, and (0 ≤ *RII* ≤ 0.2) are low [36]. 

MADM is the degree to which participants agree with each statement/question, as indicated by the average distance from the participants’ median rating [37]. MADM was applied in studies among healthcare professionals, using a Likert scale, by Hutchings et al. in 2005 [38] and Taylor et al. in 2016 [37]. In the present study, we calculated MADM using a five-point Likert scale. The MADM process involves the following: Step 1: Calculate the median of each of the 23 variables; Step 2: Calculate the absolute deviation for each variable value (x) using the formula [*xi* − *median*]; Step 3: Find the median of the absolute deviations (MAD); Step 4: Calculate the mean of the absolute deviation, also known as MADM. A median of 4–5 signifies strong support, a score of 3 signifies moderate support, and a score of 1–2 signifies low support. MADMs were measured and the values were classified into three levels of agreement, where values (<1.08) signify high agreement, (1.08–1.41) signify moderate agreement, and (>1.41) signify low agreement [37].

Analysis of variance (ANOVA) comparing the overall motivation and hospital ownership was performed. ANOVA was also performed by comparing the means of motivational outcomes between hospital ownership groups; a 5% significance level (*p* < 0.05) was applied as a cut-off point in all cases. 

To perform regression analyses, data were recoded to dichotomous (or dummy) variables for ordinal data, excluding continuous variables (see Table 1). 

Multivariate regression analyses were performed to identify the statistically significant factors of healthcare professionals’ motivation. Motivation (*Y_i_*) was the dependent variable, and sociodemographic and work environment factors were independent variables (*X_ji_*). The linear multivariate regression model estimated in this study is represented below [39]:

$$Y_{i}=\beta_{0}+\beta_{1}X_{1i}+\beta_{2}X_{2i}+\ldots +\beta_{25}X_{25i}+\in_{i}$$

where

*β*_0_ = indicates the constant or intercept term capturing the unexplained variations in the dependent variable *Y*. 

*β*_1_ = the slope coefficient measuring the amount that *Y* will change when *X* changes by a single unit. 

i = goes from 1 to n; in this case, the 25 variables.

*X*_1*i*_ = stands for the *i*th observation value for the independent variable of *X*_1_.

∈*_i_* = is the error (disturbance) term that represents other factors that affect motivation but are not explicitly considered in the model.

Student’s *t*-test was used to determine whether each independent variable regression slope coefficient was statistically significant at a 95% level of confidence. A two-tailed *t*-test was applied to the nondirectional hypotheses. The 5% significance level indicates that factors with *p*-values less than or equal to 0.05 are regarded as statically significant. 

### 2.4. Ethical Considerations

Three institutions approved the study protocol. The initial approval was from the Faculty of Health Sciences Research Ethical Committee, University of Pretoria, South Africa (approval number 718/2019). The subsequent approval was in Kenya from the Institutional Review Board, United States International University, Africa (ethical approved number USIU-A/IRB/130-2020). At the national level, approval to carry out this study was granted by the National Commission for Science, Technology, and Innovation (NACOSTI; research license number NACOSTI/P/20/4133). Approval was received from the Department of Health, Meru county Government (approval number CGM/COH/1/17(50)). Administrative approval from all the 24 hospitals was obtained, and informed consent forms were signed by the individuals who voluntarily participated in this study.

## 3. Results

A total of 553 healthcare professionals from various cadres participated in this study. 

### 3.1. Sociodemographic Characteristics

Participants’ median age was 35 years, with a range of 20 to 78 years. Three-quarters (74.5%) worked in public hospitals. Females accounted for 61.3%, and 63.1% of participants were married. Among the 553 participants, 60.6% were diploma holders, 93.3% were employed full-time, and 66% had received in-service training. On average, participants had 10 years of professional experience and worked 39 h per week. Approximately 48.2% of the participants earned 65,000 Kenyan Shillings (KES) (USD 650) or less per month, and the median household size was three people. Most (86.62%) of the participants were not offered staff housing. Of the 13.38% who were offered staff housing, 11.57% lived in permanent-type structures (stone or concrete), 1.63% were in semipermanent housing (steel or wooden walls and concrete floor), and 0.8% were offered temporary housing (a hut or mud house).

The skill mix represented among the participants included nursing professionals (30.56%), clinical officers (18.08%), medical laboratory scientists (9.76%), dentistry professionals (9.40%), pharmaceutical professionals (7.41%), public health specialists (7.23%), physicians or specialist doctors (5.61%), health records officers (3.62%), nutrition and dietetic specialists (3.07%), physiotherapists (2.17%), radiologists (2.17%), and mental health specialists (0.90%).

### 3.2. Motivation Outcomes 

Table 2 presents the mean and standard deviation (*SD*) scores for each motivation outcome/construct depicted in three categories: overall, public hospitals, and mission hospitals. The order from the highest-ranked motivator to the lowest was conscientiousness, timeliness and attendance, intrinsic job satisfaction, job satisfaction, general motivation, burnout, and organizational commitment. In public hospitals, participants’ mean scores for conscientiousness were the highest and organizational commitment was the lowest, while in mission hospitals, the mean scores for timeliness and attendance were the highest and the organizational commitment score was the lowest. Overall, participants in both public and mission hospitals had the highest mean scores for conscientiousness and lowest for organization commitment. The details of the mean, median, and standard deviation scores for each question can be found in Table S1.

### 3.3. Relative Importance Index

The motivation outcomes were ranked according to their RIIs. Table 3 illustrates the RIIs and rank of the motivation constructs/outcomes among the participants. Five motivational outcomes had high–medium relative importance, which, in order of highest to lowest, were conscientiousness, organizational commitment, intrinsic job satisfaction, job satisfaction, and general motivation. Burnout held the sixth position with medium relative importance, and timeliness and attendance were in the seventh position with medium–low relative importance. Details on RIIs for all the questions can be found in Table S2. 

### 3.4. Mean Absolute Deviation from the Median (MADM) 

Table 4 shows a high agreement with 91.3% (21/23) of the questions. This is depicted in the strong support of median scores between 4 and 5 on the 5-point Likert scale. The remaining two statements, “I do this job as it provides long-term security for me,” and “I feel very little commitment to this hospital,” had moderate and low support shown in median scores, at 3 and 2, respectively. The strength of agreement was high for 95.7% (22/23) of questions (see Table 4). However, the strength of agreement was low (*MADM* = 1.54) for one statement: “Sometimes when I get up in the morning, I dread having to face another day at work”.

### 3.5. ANOVA Results

The ANOVA results showed that hospital ownership had no statistically significant impact on the overall motivation of healthcare professionals in this study (*p* = 0.516). Approximately 0.08% (0.10% adjusted *R*^2^, *F* = 0.43, *df* = 552, *p* = 0.511) of the variation in the overall motivation of healthcare professionals was explained by hospital ownership.

Table 5 demonstrates that five motivational outcomes had significant differences between public and mission hospitals’ mean ratings, namely, burnout, job satisfaction, intrinsic job satisfaction, conscientiousness, and timeliness and attendance. However, two motivational outcomes showed statistically nonsignificant differences in the mean ratings between public and mission hospitals, namely, general motivation and organizational commitment. 

### 3.6. Multivariate Regression

The coefficient of determination (*R*^2^) of 0.2995 implies that the sociodemographic and work-environment variables included in the linear multivariate regression model explained 29.95% (26.63% if adjusted *R*^2^, *F* = 9.01, *p* < 0.001) of the variation in the participants’ overall motivation. 

Table 6 shows the results of the multivariate regression model, where six independent variables were statistically significant, namely, household size, occurrence of water unavailability, safe drinking water, acceptable main source of water, handwashing station ≤5 m from toilets, and overall safety of the hospital environment. Seven sociodemographic variables had negative coefficients, but all were statistically nonsignificant: age, income, marital status, qualifications, HRH professional cadre, type of employment, and hours worked per week. Age, for example, was *r* = −0.070, meaning that as healthcare professionals’ age increases, the less motivated they report being. Four work environment variables had negative coefficients: consistent supply of water, occurrence of water unavailability, safe drinking water, and risk when using toilet facilities. However, two variables were statistically significant: occurrence of unavailable water and safe drinking water. The other two work-environment independent variables (consistent supply of water and risk when using toilet facilities) were statistically nonsignificant. 

## 4. Discussion

Healthcare professionals’ motivation has been considered important in healthcare policy development. Using the types of motivation presented in SDT, namely, intrinsic and well-internalized extrinsic factors, results in autonomous motivation and hence reduces the probability of amotivation [21]. The present research will enhance the understanding of motivation levels and factors among healthcare professionals in a rural context, specifically, Meru county in Kenya.

In this study, participants’ overall motivation score was 75.55%. This is slightly higher than the scores reported in previous studies on Ethiopia [33] and Gaza [40], with overall motivation scores of 58.6% and 66.2%, respectively. 

This study found high levels of agreement across the motivational outcomes among healthcare professionals in public and mission hospitals. However, within the motivation outcomes (subscales), some were significantly different between professionals at public versus mission hospitals. The motivational outcomes, in order of relative importance, were conscientiousness, organizational commitment, intrinsic job satisfaction, job satisfaction, general motivation, burnout, and timeliness and attendance. 

Conscientiousness is an intrinsic motivation outcome that was significantly different between healthcare professionals at public and mission hospitals. In this study, the respondents in public hospitals reported higher conscientiousness than those in mission hospitals. In this study, conscientiousness was the highest in terms of relative importance; conscientiousness refers to the tendency of an individual to follow norms and rules and exercise self-discipline by working on achieving organizational goals [41]. Similar findings were reported in Ethiopia, where conscientiousness was the highest dimension, with a mean of 4.06 [33]. Self-perceived conscientiousness among health workers in the public sector of Ethiopia was reported as being statistically significant [42]. Another study among healthcare employees in the private healthcare sector in India reported conscientiousness exerted a significant buffering effect against burnout [43]. This finding implies that healthcare professionals in this study and other studies perceive conscientiousness as a motivational outcome that positively impacts their motivation to work effectively.

Organizational commitment is an intrinsic motivational outcome and was second highest in terms of relative importance among healthcare professionals. Organizational commitment is the extent to which an individual intrinsically adopts, identifies with, and is actively involved in their work to achieve organizational goals [44]. Our findings show no significant difference in organizational commitment based on hospital ownership. Franco and colleagues [14] stated that organizational commitment, also known as job commitment, can be increased through transformational leadership by ensuring the goals of the organization (e.g., hospitals) are personally relatable to the healthcare professionals, resulting in their increased motivation. Lack of organizational commitment could potentially result in poor implementation of crucial healthcare system reforms due to lack of motivation and can be attributed to incongruence in values and goals between healthcare professionals and their organization [14].

In this study, job satisfaction was an extrinsic motivation outcome defined as the overall degree of contentment a healthcare professional feels based on external factors relating to their work. A study in Portugal reported that job satisfaction positively correlated with all motivation factors and was a critical element of healthcare service delivery among healthcare workers [45]. The current study found that intrinsic job satisfaction was relatively more important than job satisfaction among the participants. Additionally, a Cyprus study reported that the highest motivator among doctors and nurses was achievement, characterized as an intrinsic factor involving the recognition of positive behavior and opportunities for career growth [46]. A cross-sectional design study in India found that medical officers ranked intrinsic factors related to motivation to be of higher importance than extrinsic factors of motivation [47]. Both job satisfaction and intrinsic job satisfaction were significantly different between public and mission hospitals, with healthcare professionals in mission hospitals reporting a higher job satisfaction mean score than those in public hospitals, at 3.80 versus 3.86, respectively. The opposite was true regarding intrinsic motivation, with public hospitals reporting a higher mean of 4.17, compared to 4.09 in mission hospitals.

Burnout among healthcare professionals can be attributed to challenges such as shortage of personnel, excess workload, and lack of support [14]. These are challenges that can result in chronic stress and fatigue that can be depicted through the three dimensions of emotional exhaustion, depersonalization, and inefficacy [48]. In this study, participants perceived burnout as the sixth most important motivational outcome. Burnout significantly varied among healthcare professionals in public and mission hospitals, with mean scores of 3.33 and 3.46, respectively; those in mission hospitals experienced more burnout. In India, burnout was reported as a demotivator among health workers working in rural health facilities [49]. Thus, health policymakers need to develop motivation strategies and interventions geared toward empowering healthcare professionals to cope with stress in a healthy and proactive manner to avoid burnout. 

Timeliness and attendance are extrinsic motivational outcomes associated with punctuality and being present in the workplace during the required time. In this study, timeliness and attendance had a high mean score and were of the lowest relative importance to overall motivation among the participants. However, timeliness and attendance were significantly different between the public and mission hospitals, with professionals from mission hospitals having a higher mean score than those from public hospitals (4.39 versus 4.16, respectively). Similarly, in Zambia, high scores for the timeliness motivation outcome were indicated by healthcare workers in rural health facilities [34]. In Ethiopia, moderate scores of 3.56 were reported among healthcare workers [33]. Our results imply that healthcare professionals are motivated to keep time and be present at work, although it may not have been the highest RII.

Six predictors of healthcare professionals’ overall motivation were revealed in this study: household size, the occurrence of water unavailability, safe drinking water, acceptable main source of water, handwashing station ≤5 m from toilets, and overall safety of the hospital environment. 

Household size was a significant extrinsic predictor of healthcare professionals’ overall motivation; the larger the household size, the more motivated they were. This could be attributed to the larger responsibility stemming from the high number of dependents in their family [11]. Conversely, marital status was a negative predictor of overall motivation among the participants, implying that participants who were married were less motivated than those who were single, divorced, or widowed, but the results were nonsignificant. This finding is in line with a study in rural Tanzania that reported marital status as a significant predictor of healthcare workers’ motivation, with married health workers being less motivated than their single, widowed, or separated colleagues [50]. Similarly, in Uganda, health workers who were single or separated reported higher motivation compared to their married colleagues [51]. These results imply that household size could be a well-internalized extrinsic motivator contributing to autonomous motivation in African nations. The opposite could be true for marital status, as marriage was associated with lower motivation scores, implying the possibility of reduced autonomous motivation and more controlled motivation.

Work environment is a significant extrinsic motivation factor in healthcare professionals’ motivation in Africa [52]. A study in Tanzania reported a poor work environment as the second-highest demotivator among healthcare workers, especially among clinically-trained professionals [53]. In India, public and private health workers rated a good work environment as more important than good income [54]. In this study, the unavailability of water in the hospital significantly decreased healthcare professionals’ motivation; a perceived acceptable primary source of water (in terms of color, odor, and taste) resulted in increasing overall motivation. Furthermore, the closer the handwashing station was to the toilets, the more motivated the healthcare professionals were. The existence of formal hospital waste disposal systems also increased overall motivation. 

The participants reported that the higher the perception of a safe hospital working environment, the higher their overall motivation. Our findings were concurrent with a study in Benin and Kenya, in which healthcare professionals reported a good working environment is a significant factor in their motivation [52]. Similarly, in Jordan, a cross-sectional study among 582 registered nurses indicated that the work environment was positively related to a nurse’s motivation to stay [55]. On the contrary, a poor working environment/condition led to demotivation among the healthcare workforce [52]; the same was confirmed in a study in Cyprus [46]. Our findings imply that, although the work environment is an extrinsic factor, the healthcare professionals in our study perceive it as an enabler of achieving organizational goals. This indicates that the work environment is an example of a well-internalized extrinsic motivator [20,21] among the participants in this study. Therefore, improving the work environment can be a strategy for simultaneously increasing healthcare professionals’ autonomous motivation and quality of care. 

In Ethiopia, the type of hospital is an extrinsic factor that has been strongly associated with increased intrinsic motivation [33]; however, hospital ownership or type of hospital was a statistically nonsignificant predictor of healthcare professionals’ motivation in this study. Our results imply that there was no significant difference between participants’ overall motivation in public and mission hospitals, suggesting that similar policies and strategies to increase motivation can be applied to both settings. However, comparing the results from the Ethiopian study with the present research also shows the need for investigating the role of hospital ownership in multiple settings to capture similarities or differences. 

The six predictors of healthcare professionals’ motivation revealed in this study are related to their family, health, and safety. The findings on the predictors and motivational outcomes show the role of intrinsic and well-internalized extrinsic factors on the overall motivation of the participants. Based on SDT, our results contribute to the knowledge regarding the role of intrinsic and well-internalized extrinsic factors in a rural health care setting. Our research findings suggest that healthcare professionals primarily strive for autonomous motivation, which is a potential focus for motivation interventions. Both intrinsic and well-internalized extrinsic factors are key for autonomous motivation and positively impact psychological growth, mastery, and connectedness, which are key to enhancing and maintaining motivation at work [20,21]. 

## 5. Conclusions

In the Kenyan context, this is the first study to investigate motivation among healthcare professionals guided by SDT. This paper presents the level of motivation and type of motivation factors influencing healthcare professionals’ self-assessed motivation in public and mission hospitals in Meru county, Kenya. Their overall motivation was moderately high, regardless of the multiple challenges the healthcare workforce faces when working in a suboptimally functioning health system. The findings show that there was no significant difference between healthcare professionals’ overall motivation and hospital ownership. However, among four specific motivational outcomes, there were significant differences based on hospital ownership. The top three relatively important motivational outcomes were conscientiousness, organizational commitment, and intrinsic job satisfaction. Healthcare professionals’ overall motivation was predicted by household size and four work environment factors that are concurrent and relevant in the African context based on previous studies. Based on SDT, the well-internalized extrinsic factors and intrinsic motivational outcomes reported in this study show that both intrinsic and extrinsic factors have an impact on healthcare professionals’ motivation; thus, neither should be neglected. Specifically, these results contribute to the knowledge of factors that impact psychological growth, mastery, and connectedness, which are critical for enhancing motivation in a healthcare setting [21]. Furthermore, among the six predictors, five were majorly related to health and safety issues, showing the significance of the healthcare professionals’ health in amplifying their motivation. These findings contribute to bridging the knowledge gap and could assist county healthcare policymakers and healthcare facility managers in developing pertinent evidence-based policies and interventions aimed at increasing motivation that contribute to strengthening healthcare systems and the healthcare workforce.

## 6. Limitations

This study applied a cross-sectional design that investigated healthcare professionals’ motivation in public and mission hospitals at one point in time. Due to the study design, only correlations and strengths of relationships between variables could be reported, not causality. The questionnaire used in this study used a Likert-type scale; thus, response bias and social desirability bias may be present. To reduce these tendencies and biases, half the questions were positively worded and half were negatively worded [31]. Additionally, participants were requested to be as honest as possible, and anonymity was ensured. Selection bias could be a possible limitation because the healthcare professionals that participated could have been more motivated than those who were absent when the questionnaires were being completed. 

## 7. Areas for Future Studies

First, the Government of Meru County could fund experimental design studies to develop and evaluate the cost-effectiveness of alternative interventions for healthcare professionals’ motivation in the context of strengthening healthcare systems. Since the present study was conducted in a rural area that is relatively endowed with a good climate and fertile volcanic soils that are conducive to agriculture, the national government ought to consider sponsoring motivation studies in the semiarid and insecure counties to identify the motivation factors that could be used to attract and retain healthcare professionals in such challenging environments [5]. Such studies would yield a comprehensive nationwide understanding of factors influencing healthcare professionals’ motivation. These studies could bridge extant knowledge gaps and could both inform and transform motivational policies and interventions that are applicable and relevant to the Kenyan healthcare system.

## Figures and Tables

**Table 1:** Descriptions of sociodemographic and work environment variables.

Variable	Description
Hospital ownership (X_1_)	0 = Public hospital; 1 = Mission hospital.
Sex (X_2_)	0 = Male; 1 = Female
Age (X_3_)	Participants’ age in years.
Income per month in Kenyan Shillings (X_4_)	0 = ≤ 15,000 to 45,000; 1 = 45,999–105,999 or more.
Marital status (X_5_)	0 = Single, Divorced, or Widowed; 1 = Married
Qualifications (X_6_)	0 = Certificate and Diploma; 1 = Bachelor’s degree or higher.
Years of experience (X_7_)	Participants’ years of professional practice.
HRH professional cadre (X_8_)	0 = nonclinical healthcare professionals (medical laboratory scientists, public health specialists, nutritionists, physiotherapists, radiologists, and health records officers). 1 = clinical healthcare professionals (physicians, specialists, nurses, dentists, pharmacists, clinical officers, and mental health practitioners).
Type of employment (X_9_)	0 = Part-time; 1 = Full-time.
In-service training (X_10_)	0 = No; 1 = Yes
Hours worked per week (X_11_)	The number of hours participants work per week.
Household size (X_12_)	The number of people in the respondent’s household, including themselves.
Staff housing (X_13_)	0 = No; 1 = Yes
Consistent supply of water (X_14_)	0 = No; 1 = Yes
Occurrence of water unavailability (X_15_)	0 = No; 1 = Yes
Safe drinking water (X_16_)	0 = No; 1 = Yes
Acceptable main source of water (X_17_)	0 = No; 1 = Yes
Type of toilet facility (X_18_)	0 = Flush or pour flush; 1 = Pit latrine.
Risk when using toilet facility (X_19_)	0 = No; 1 = Yes
Hospital disposal of garbage (X_20_)	0 = Informal disposal (informal collection service, disposal in a designated area, disposal within the hospital compound, disposal elsewhere (burning, burying, or other)); 1 = Formal collection service.
Availability of soap for handwashing (X_21_)	1 = Yes; 0 = No
Constant availability of soap (X_22_)	1 = Yes; 0 = No
Handwashing station ≤5 m from the toilet (X_23_)	1 = Yes; 0 = No
Workplace safety and health committee (X_24_)	1 = Yes; 0 = No
Overall safety of hospital working environment (X_25_)	The perceived overall safety of the hospital (continuous variable).

**Table 2:** Mean scores * (SD; 1–5 Likert scale) of motivating outcomes for the sample of healthcare professionals by hospital ownership (*n* = 553).

Motivational Outcomes	Overall (*n* = 553)	Public Hospitals (*n* = 434)	Mission (Faith-Based) Hospitals (*n* = 119)
General motivation	3.38 (0.50)	3.36 (0.51)	3.44 (0.48)
Burnout	3.36 (0.05)	3.33 (0.05)	3.46 (0.06)
Job satisfaction	3.81 (0.22)	3.80 (0.18)	3.86 (0.36)
Intrinsic job satisfaction	4.16 (0.07)	4.17 (0.06)	4.09 (0.18)
Organizational commitment	3.34 (0.60)	3.34 (0.59)	3.33 (0.62)
Conscientiousness	4.37 (0.11)	4.39 (0.11)	4.30 (0.16)
Timeliness and attendance	4.21 (0.04)	4.16 (0.03)	4.39 (0.06)

* Reported on 1–5 scale where higher values are suggestive of higher motivation.

**Table 3:** Relative importance of motivation outcomes among healthcare professionals (*N* = 553).

Motivational Outcomes	Relative Importance Index	Importance
Conscientiousness	0.737	1
Organizational commitment	0.722	2
Intrinsic job satisfaction	0.689	3
Job satisfaction	0.621	4
General motivation	0.610	5
Burnout	0.529	6
Timeliness and attendance	0.358	7

**Table 4:** The median and mean absolute deviation from the median for the motivation questions (*N* = 553).

Construct	Questions	Number (%) Rating < 4	Median	MAD ^1^	MADM ^2^
General motivation	These days, I feel motivated to work as hard as I can.	198 (35.80)	4	1	0.89
	I only do this job so that I get paid at the end of the month.	210 (37.97)	4	1	0.93
	I do this job as it provides long-term security for me.	390 (70.52)	3	1	1.01
Burnout	I feel emotionally drained at the end of every day.	270 (48.82)	4	1	1.00
	Sometimes when I get up in the morning, I dread having to face another day at work.	269 (48.64)	4	2	1.54
Job satisfaction	Overall, I am very satisfied with my job.	214 (38.70)	4	1	0.86
	I am not satisfied with my colleagues in my ward.	134 (24.23)	4	1	0.85
	I am satisfied with my supervisor.	190 (34.36)	4	1	0.76
Intrinsic job satisfaction	I am satisfied with the opportunity to use my abilities in my job.	97 (17.54)	4	1	0.66
	I am satisfied that I accomplish something worthwhile in this job.	87 (15.73)	4	1	0.65
	I do not think that my work in the hospital is valuable these days.	132 (23.87)	5	0	0.89
Organizational commitment	I am proud to be working for this hospital.	153 (27.67)	4	1	0.68
	I find that my values and this hospital’s values are very similar.	247 (44.67)	4	1	0.78
	I am glad that I work for this facility rather than other facilities in the country.	266 (48.10)	4	1	0.88
	I feel very little commitment to this hospital.	477 (86.26)	2	1	0.78
	This hospital really inspires me to do my very best on the job.	247 (44.67)	4	1	0.85
Conscientiousness	I cannot be relied on by my colleagues at work.	84 (15.19)	5	0	0.63
	I always complete my tasks efficiently and correctly.	80 (14.47)	4	1	0.66
	I am a hard worker.	49 (8.86)	5	0	0.50
	I do things that need doing without being asked or told.	68 (12.30)	5	0	0.63
Timeliness and attendance	I am punctual about coming to work.	93 (16.82)	4	1	0.69
	I am often absent from work.	109 (19.71)	5	0	0.75
	It is not a problem if I sometimes come late to work.	114 (20.61)	5	0	0.82

^1^ MAD: median absolute deviation. ^2^ MADM: mean absolute deviation from the median. Questions were rated on a five-point Likert scale, from strongly disagree to strongly agree for positively worded questions, and strongly agree to strongly disagree for negatively worded questions. Median ≥4 indicates high agreement.

**Table 5:** Motivation outcomes ANOVA by hospital ownership.

Motivation Outcome	Hospital Ownership	Mean	*SD*	*n*	*F*	*p*
General motivation	Public	3.36	0.51	434	2.356	0.125
	Mission	3.44	0.48	119		
Burnout	Public	3.33	0.05	434	576.966	0.001 *
	Mission	3.46	0.06	119		
Job satisfaction	Public	3.80	0.18	434	6.318	0.012 *
	Mission	3.86	0.36	119		
Intrinsic job satisfaction	Public	4.17	0.06	434	61.193	0.001 *
	Mission	4.09	0.18	119		
Organizational commitment	Public	3.34	0.59	434	0.026	0.871
	Mission	3.33	0.62	119		
Conscientiousness	Public	4.39	0.11	434	50.462	0.001 *
	Mission	4.30	0.16	119		
Timeliness and attendance	Public	4.16	0.03	434	3342.164	0.001 *
	Mission	4.39	0.06	119		

Reported on a 5-point Likert scale, with higher values suggesting higher motivation. * *p* < 0.05 indicates statistical significance.

**Table 6:** Multivariate regression of overall motivation, sociodemographic, and work-environment factors (*n* = 553).

Overall Motivation	Coef.	Standard Error	*t*-Value	*p*	Beta
Hospital ownership	0.040	1.060	0.04	0.970	0.002
Sex	1.175	0.824	1.43	0.154	0.054
Age	−0.070	0.078	−0.89	0.372	−0.063
Income	−1.658	1.055	−1.57	0.117	−0.069
Marital status	−1.198	0.938	−1.28	0.202	−0.055
Qualification	−1.595	0.881	−1.81	0.071	−0.071
Years of experience	0.035	0.090	0.39	0.700	0.027
HRH professional cadre	−0.864	0.910	−0.95	0.342	−0.037
Type of employment	−0.023	1.677	−0.01	0.989	−0.001
In-service training	0.364	0.888	0.41	0.682	0.016
Hours worked per week	−0.010	0.030	−0.34	0.732	−0.013
Household size	0.610	0.247	2.47	0.014 *	0.110
Staff housing	0.182	1.214	0.15	0.881	0.006
Consistent supply of water	−0.329	1.279	−0.26	0.797	−0.012
Occurrence of water unavailability	−3.858	0.865	−4.46	<0.001 *	−0.178
Safe drinking water	−2.740	1.060	−2.58	0.010 *	−0.112
Acceptable main source of water	5.316	1.261	4.21	<0.001 *	0.187
Type of toilet facility	1.524	0.953	1.60	0.110	0.061
Risk when using toiletry facility	−0.164	0.969	−0.17	0.866	−0.007
Hospital disposal of garbage	1.734	1.034	1.68	0.094	0.063
Availability of water for handwashing	0.912	1.427	0.64	0.523	0.031
Constant availability of soap	3.049	1.701	1.79	0.074	0.079
Handwashing station ≤5 m from the toilet	4.840	1.591	3.04	0.002 *	0.135
Workplace safety and health committee	1.263	0.912	1.38	0.167	0.059
Overall safety of hospital working environment	1.303	0.233	5.60	<0.001 *	0.238
*Constant*	70.150	3.488	20.11	<0.001	

* *p* < 0.5 indicates statistical significance.

## Supplementary Materials

The following are available online at https://www.mdpi.com/2227-9032/8/4/530/s1. Table S1: Motivation outcomes and 23 questions with the median, mean and standard deviation scores (n = 553). Table S2: Relative importance indices for all 23 questions (n = 553).

## Appendix A

**Table A1:** Reliability coefficients of the motivational subscales.

Motivational Subscales	Reliability Coefficient
General motivation	0.348
Burnout	0.570
Job satisfaction	0.399
Intrinsic job satisfaction	0.588
Organizational commitment	0.786
Conscientiousness	0.649
Timeliness and attendance	0.611
Overall Cronbach’s alpha (internal consistency)	0.851
