# Supplementary material for: An Investigation of Healthcare Professionals’ Motivation in Public and Mission Hospitals in Meru County, Kenya
Source: Healthcare (Basel). 2020 Dec 2;8(4):530. doi: 10.3390/healthcare8040530 (PMC7761626; doi:10.3390/healthcare8040530)
Supplement: Supplementary file 1 [file healthcare-08-00530-s001.pdf]

An investigation of healthcare professionals' motivation in public and mission hospitals in Meru County, Kenya.

Supplementary file 1: Detailed tables of Frequencies, percentages and relative importance indices of all the 23 questions.

Authors

Rose Nabi Deborah Karimi Muthuri*[1], Flavia Senkubuge[1] and Charles Hongoro[1,2,3,4].

Authors affiliation

[1] School of Health Systems and Public Health (SHSPH), Faculty of Health Sciences, University of Pretoria, Pretoria, South Africa.

[2] Developmental, Capable and Ethical State Division, Human Sciences Research Council (HSRC), Pretoria, South Africa.

[3] Faculty of Science, Tshwane University of Technology, Tshwane, South Africa.

[4] Faculty of Health Sciences, Fort Hare University, Alice, South Africa.

*Corresponding author: Rose Nabi Deborah Karimi Muthuri (**Email:**u19391189@tuks.co.za)

Table S1. Motivational outcomes and 23 questions with median, mean and standard deviation scores

Motivational outcome	Questions	Median	Mean scores (5-point Likert Scale)	Standard deviation
General motivation	These days, I feel motivated to work as hard as I can.	4	3.65	1.20
	I only do this job so that I get paid at the end of the month.	4	3.69*	1.21
	I do this job as it provides long term security for me.	3	2.80*	1.22
Burnout	I feel emotionally drained at the end of every day.	4	3.32*	1.20
	Sometimes when I get up in the morning, I dread having to face another day at work.	4	3.39*	1.20
Job satisfaction	Overall, I am very satisfied with my job.	4	3.65	1.13
	I am not satisfied with my colleagues in my ward.	4	4.06*	1.10
	I am satisfied with my supervisor.	4	3.73	1.07
Intrinsic job satisfaction	I am satisfied with the opportunity to use my abilities in my job.	4	4.12	0.94
	I am satisfied that I accomplish something worthwhile in this job.	4	4.24	0.84
	I do not think that my work in the hospital is valuable these days.	5	4.11*	1.18
Organizational commitment	I am proud to be working for this hospital.	4	3.92	0.97
	I find that my values and this hospital's values are very similar.	4	3.50	1.00
	I am glad that I work for this facility rather than other facilities in the country.	4	3.44	1.08
	I feel very little commitment to this hospital.	2	2.33*	1.04
	This hospital really inspires me to do my very best on the job.	4	3.30	1.09
Conscientiousness	I cannot be relied on by my colleagues at work.	5	4.37*	1.05
	I always complete my tasks efficiently and correctly.	4	4.23	0.88
	I am a hard worker.	5	4.50	0.73
	I do things that need doing without being asked or told.	5	4.37	0.85
Timeliness and attendance	I am punctual about coming to work.	4	4.20	0.92
	I am often absent from work.	5	4.25*	1.10
	It is not a problem if I sometimes come late to work.	5	4.18*	1.04

* The scale for negatively worded questions was reversed thus, 1 (strongly agree) to 5(strongly disagree). Therefore, a higher score shows disagreement with negative statement and thus, implying higher motivation.

Table S2. Relative importance indices for all 23 motivation questions

Question		Strongly Agree	Agree	Neutral	Disagree	Strongly Disagree	Total	Total number	A*N	RII	Rank
1.	These days, I feel motivated to work as hard as I can.	745	824	273	136	39	2017	553	2765	0,729	10
2.	I only do this job so that I get paid at the end of the month.	205	200	357	342	172	1276	553	2765	0,461	17
3.	I do this job as it provides long term security for me.	420	664	420	208	59	1771	553	2765	0,641	14
4.	I feel emotionally drained at the end of every day.	250	384	372	388	89	1483	553	2765	0,536	15
5.	Sometimes when I get up in the morning, I dread having to face another day at work.	200	392	393	344	112	1441	553	2765	0,521	16
6.	Overall, I am very satisfied with my job.	700	796	375	118	30	2019	553	2765	0,730	9
7.	I am not satisfied with my colleagues in my ward.	85	204	198	336	251	1074	553	2765	0,388	18
8.	I am satisfied with my supervisor.	670	916	366	80	28	2060	553	2765	0,745	7
9.	I am satisfied with the opportunity to use my abilities in my job.	1075	964	165	62	11	2277	553	2765	0,824	5
10.	I am satisfied that I accomplish something worthwhile in this job.	1270	884	204	26	6	2390	553	2765	0,864	3
11.	I do not think that my work in the hospital is valuable these days.	125	196	174	258	292	1045	553	2765	0,378	19
12.	I am proud to be working for this hospital.	830	936	327	60	14	2167	553	2765	0,784	6
13.	I find that my values and this hospital's values are very similar.	385	916	489	122	23	1935	553	2765	0,700	12
14.	I am glad that I work for this facility rather than other facilities in the country.	455	784	495	140	31	1905	553	2765	0,689	13
15.	I feel very little commitment to this hospital.	620	876	402	112	20	2030	553	2765	0,734	8
16.	This hospital really inspires me to do my very best on the job.	490	832	465	118	33	1938	553	2765	0,701	11
17.	I cannot be relied on by my colleagues at work.	90	128	102	228	355	903	553	2765	0,327	23
18.	I always complete my tasks efficiently and correctly.	1235	904	156	36	10	2341	553	2765	0,847	4
19.	I am a hard worker.	1710	648	111	20	2	2491	553	2765	0,901	1
20.	I do things that need doing without being asked or told.	1530	716	132	34	7	2419	553	2765	0,875	2
21.	I am punctual about coming to work.	45	108	171	428	246	998	553	2765	0,361	21
22.	I am often absent from work.	115	116	171	244	322	968	553	2765	0,350	22
23.	It is not a problem if I sometimes come late to work.	55	180	174	320	279	1008	553	2765	0,365	20
